# Role of Doping Effect and Chemical Pressure Effect Introduced by Alkali Metal Substitution on 1144 Iron-Based Superconductors

**DOI:** 10.3390/ma16093343

**Published:** 2023-04-24

**Authors:** Yi-Na Huang, Xiang-Long Yu, Da-Yong Liu, Miao-Miao Han

**Affiliations:** 1 Department of Physics, School of Science, Zhejiang University of Science and Technology, Hangzhou 310023, China; 2 Shenzhen Institute for Quantum Science and Engineering, Southern University of Science and Technology, Shenzhen 518055, China; 3 International Quantum Academy, Shenzhen 518048, China; 4 Guangdong Provincial Key Laboratory of Quantum Science and Engineering, Southern University of Science and Technology, Shenzhen 518055, China; 5 Department of Physics, School of Sciences, Nantong University, Nantong 226019, China; 6 School of Science, Huzhou University, Huzhou 313000, China

**Keywords:** iron-based superconductor, electronic structure, magnetism

## Abstract

Ca*A*Fe${}_{4}$As${}_{4}$ with *A* = K, Rb, and Cs are close to the doped 122 system, and the parent material can reach a superconducting transition temperature of 31–36 K without doping. To study the role of alkali metals, we investigated the induced hole doping and chemical pressure effects as a result of the introduction of alkali metals using density-functional-based methods. These two effects can affect the superconducting transition temperature by changing the number of electrons and the structure of the FeAs conductive layer, respectively. Our study shows that the $d_{xz}$ and $d_{yz}$ orbitals, which are degenerate in CaFe${}_{2}$As${}_{2}$, become nondegenerate in Ca*A*Fe${}_{4}$As${}_{4}$ due to two nonequivalent arsenic atoms (As1 and As2). The unusual oblate ellipsoid hole pocket at Γ point in Ca*A*Fe${}_{4}$As${}_{4}$ results from a divalent cation Ca^2^+ replaced by a monovalent cation *A*^+^. It shows one of the main differences in fermiology compared to a particular form of CaFe${}_{2}$As${}_{2}$ with reduced 1144 symmetry, due to the enhancement of As2-Fe hybridization. The unusual band appears in CaFe${}_{2}$As${}_{2}$ (1144) and gradually disappears in the change of K to Cs. Further analysis shows that this band is contributed by As1 and has strong dispersion perpendicular to the FeAs layer, suggesting that it is related to the peculiar van Hove singularity below the Fermi level. In addition, various aspects of CaFe${}_{2}$As${}_{2}$ (1144) and Ca*A*Fe${}_{4}$As${}_{4}$ in the ground state are discussed in terms of the influence of hole doping and chemical pressure.

## 1. Introduction

Since the discovery of iron-based superconductors, much attention has been paid to increasing the superconducting transition temperature $T_{c}$ and explaining the superconducting pairing mechanism, including electron–phonon pairing, *s*-wave, and *d*-wave pairing, etc. Nowadays, many families have been found, such as 11-type FeSe [1], 1111-type LaO${}_{1-x}$F${}_{x}$FeAs [2] and CaHFeAs [3], 122-type (Ba,K)Fe${}_{2}$As${}_{2}$ [4], 111-type LiFeAs [5], 112-type CaFeAs${}_{2}$ [6,7], etc. Many of them must be doped or pressured to be superconducting, except some, such as LiFeAs, which is an intrinsic superconductor without doping. It remains a challenge to control the actual doping level and pressure strength during the synthesizing process of these materials.

**Figure 1:**
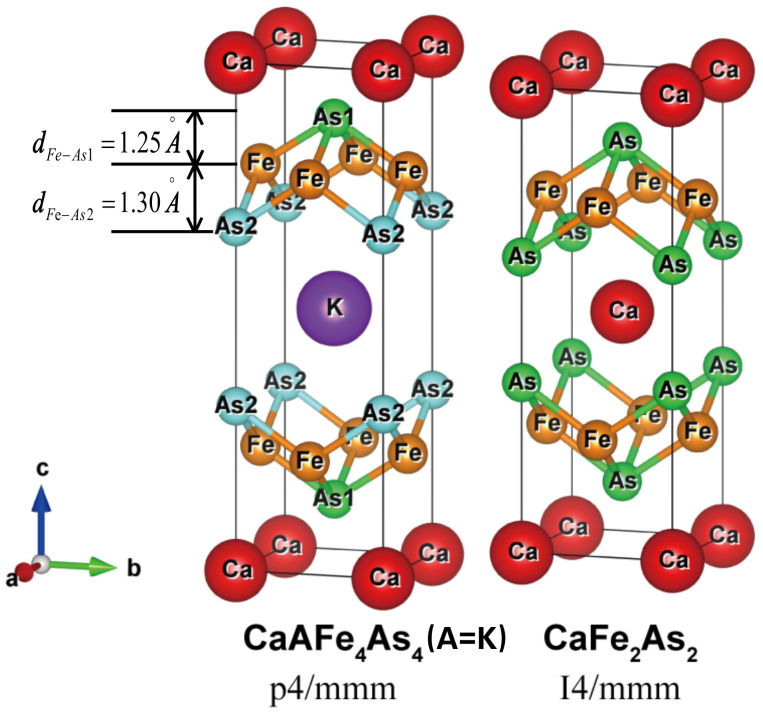
The structure of 1144-type Ca*A*Fe${}_{4}$As${}_{4}$ and 122-type CaFe${}_{2}$As${}_{2}$. Note that the tetragonal cell shown here is a primitive cell for 1144, but a conventional cell for 122.

Recently, a relatively new family of 1144-type iron-based superconductors was synthesized with a T${}_{c}$ as high as 31 K to 36 K without doping or pressure [8]. This stoichiometry of the material is valuable for studying superconducting mechanisms without the disorder. The new 1144 type includes Ca*A*Fe${}_{4}$As${}_{4}$ (*A* = K, Rb, and Cs) and Sr*A*Fe${}_{4}$As${}_{4}$ (*A* = Rb and Cs). Ca*A*Fe${}_{4}$As${}_{4}$ (spacegroup *P4/mmm*) has a different but related crystal structure compared to CaFe${}_{2}$As${}_{2}$ (spacegroup *I4/mmm*) in the nonmagnetic (NM) phase. Ca*A*Fe${}_{4}$As${}_{4}$ (*A* = K, Rb, and Cs) can be viewed as if the Ca layers in CaFe${}_{2}$As${}_{2}$ were alternately replaced by *A* layers. The alternative Ca and *A* layers cause the up and down As layer in FeAs to be layer-inequivalent, as shown in Figure 1. A number of experimental results on thermodynamic and transport properties [9], angle-resolved photoemission spectroscopy (ARPES) [10], nuclear magnetic resonance (NMR) [11], vortices [12,13], pressure effect [14], and topological aspect [15], as well as theoretical calculations [16,17,18], have been performed on Ca*A*Fe${}_{4}$As${}_{4}$ to explain their various physical properties [18]. The influence of alkali metal replacement on the FeAs layer of the material has two effects: one is doping and the other is chemical pressure.

Mei et al. revealed that the nonmagnetic state is the ground state of the studied superconductors in Ca*A*Fe${}_{4}$As${}_{4}$ [17]. Mou et al. found that the values of Ca*A*Fe${}_{4}$As${}_{4}$ superconducting gaps are nearly isotropic (within the explored portions of the Brillouin zone), but are significantly different for each of the Fermi surface (FS) sheets [10]. Using NMR studies, a new iron-based superconductor, CaKFe${}_{4}$As${}_{4}$, with T${}_{c}$ = 35 K was obtained by Cui et al. [11]. The new-structure-type Fe-based superconductors Ca*A*Fe${}_{4}$As${}_{4}$ (*A* = K, Rb, and Cs) and Sr*A*Fe${}_{4}$As${}_{4}$ (*A* = Rb and Cs) can be regarded as hybrid phases between *Ae*Fe${}_{4}$As${}_{4}$ (*Ae* = Ca and Sr) and Ca*A*Fe${}_{4}$As${}_{4}$, as found by Iyo et al. [8]. Song et al. predicted that the 1144-type phase could be stabilized in EuKFe${}_{4}$As${}_{4}$, EuRbFe${}_{4}$As${}_{4}$, EuCsFe${}_{4}$As${}_{4}$, CaCsFe${}_{4}$P${}_{4}$, SrCsFe${}_{4}$P${}_{4}$, BaCsFe${}_{4}$P${}_{4}$, InCaFe${}_{4}$As${}_{4}$, InSrFe${}_{4}$As${}_{4}$, etc [19]. Borisov et al. found in *AeA*Fe${}_{4}$As${}_{4}$ with *Ae* = Ca, Sr, and Ba and *A* = K, Rb, and Cs systems the appearance of two consecutive half-collapsed tetragonal transitions at pressures $P_{c1}$ and $P_{c2}$, which have a different character in terms of their effect on the electronic structure [20]. Liu et al. identified Dirac surface states and Majorana zero modes, respectively, for the first time in an iron-pnictide superconductor, CaKFe${}_{4}$As${}_{4}$ [15]. The anisotropic properties of wavelengths λ are explained by the multigap nature of superconductivity in CaKFe${}_{4}$As${}_{4}$ and caused by anisotropic contributions of various bands to the in-plane and the out-of-plane components of the superfluid density. This was considered by Berlijn et al. [12]. Singh (SS) indicated that the formation of phase-pure 1144 occurs over a much narrower window and is highly prone to multiphase formation, as compared with the 122 family [21]. The extraordinary vortex pinning properties in CaKFe${}_{4}$As${}_{4}$ (CaK1144) arising from the inherent defect structure were found by Ishida et al. [22]. However, some work has been carried out in theory, but only the properties of electronic state and magnetism have been studied, and no qualitative research has been carried out on the role of the two effects caused by the substitution of alkali metals and their contribution.

The role of the two effects caused by alkali metal substitution raises the following questions: First of all, what is the effect of the hole doping caused by the positive one-valent alkali metal replacing the positive two-valent calcium ion on the FeAs electron layer of the 1144 iron-based superconductor? Second, what is the effect of the chemical pressure caused by various ionic radii on the FeAs layer? Finally, what is the contribution of these two effects?

In this paper, a qualitative study of these two effects is achieved by comparing K, Rb, and Cs substitution and CaFe${}_{2}$As${}_{2}$ in 1144 P4/mmm symmetry. The first-principles approach is described in Section 2, for systematic presentation of the results of first-principle calculations of the electronic properties of Ca*A*Fe${}_{4}$As${}_{4}$ (*A* = K, Rb, and Cs) materials. Especially, we perform a special treatment of CaFe${}_{2}$As${}_{2}$ to compare its electronic structure to that of Ca*A*Fe${}_{4}$As${}_{4}$. Section 3 contains the main results. Although CaFe${}_{2}$As${}_{2}$ has a structure similar to that of Ca*A*Fe${}_{4}$As${}_{4}$, it has a different unit cell geometry because of the difference in space group, so, compared to the direct comparison of the electronic structures in two different unit cells as performed by others [17], our contrastive method is more meaningful. Therefore, we intentionally mark the Ca and As atoms in CaFe${}_{2}$As${}_{2}$ nonequivalently the same as Ca*A*Fe${}_{4}$As${}_{4}$, then performed the first-principle calculation for CaFe${}_{2}$As${}_{2}$ under the same tetragonal unit cell and P4/mmm symmetry as Ca*A*Fe${}_{4}$As${}_{4}$. In this way, we obtain several meaningful comparisons of the band structure, Fermi pockets, and their orbital projections between these two series of iron superconductors. We also calculate the general susceptibility, which reflects the geometrical nesting of electron and hole pockets and the total energy of different magnetic ordering structures. A discussion and synopsis follow in Section 4.

## 2. Materials and Methods

### 2.1. Crystal Structure

The structure of 1144-type Ca*A*Fe${}_{4}$As${}_{4}$ (*A* = K, Rb, and Cs) is quite similar to 122-type CaFe${}_{2}$As${}_{2}$ with 50% of Ca replaced by alkali atoms, as shown in Figure 1. However, due to the drastic difference in ionic radii, K, Rb, and Cs atoms cannot occupy the crystallographically equivalent 122 Ca site, leading to a separate Ca and *A* layers, and changing the space group from *I4/mmm* to *P4/mmm*. The separation of the Ca and *A* layers makes 1144 effectively a stoichiometric hole doping phase 122. Moreover, the two As layers that sandwich the Fe layer become nonequivalent due to the periodic arrangement of the Ca and *A* layer. We label two nonequivalent As as As1 (near the Ca layer) and As2 (near the *A* layer), and As1 is closer to the Fe plane than As2. Atom *A* also separates FeAs layers greater than Ca atom due to larger ionic radii and causes a larger *c* value of the unit cell.

Except for the quite similar crystal structure, CaFe${}_{2}$As${}_{2}$ is a nonsuperconducting material, so it is our interest to compare electronic structures between 1144 and 122. However, CaFe${}_{2}$As${}_{2}$ has an *I4/mmm* symmetry, resulting in a primitive cell that is nontetragonal, unlike that of 1144. Fortunately, the conventional cell of CaFe${}_{2}$As${}_{2}$ is the same as the primitive cell of Ca*A*Fe${}_{4}$As${}_{4}$. We intentionally label two Ca and two As atoms in CaFe${}_{2}$As${}_{2}$ conventional cells nonequivalently the same as Ca*A*Fe${}_{4}$As${}_{4}$, and generate a structure in 1144 P4/mmm symmetry. We calculate the electronic properties of these artificial CaFe${}_{2}$As${}_{2}$, which we will call CaFe${}_{2}$As${}_{2}$ (1144) later on.

We used lattice parameters and optimized atomic positions of Ca*A*Fe${}_{4}$As${}_{4}$ from Suetin et al. [23], and the structural parameters are shown in Table 1.

### 2.2. Calculational Methods

We used the Wien2K package [24], which is based on the augmented plane wave plus local orbitals (APW+lo) method, for the electronic structure calculations and the Perdew–Burke–Emzerhof (PBE) [25] version of the generalized gradient approximation (GGA) within density functional theory. The sphere radii for Ca, K, Rb, and Cs were taken as 2.3 bohr, and those for Fe and As were taken as 2.21 and 2.11 bohr, respectively. The convergence of this basis set cut-off parameter $R_{mt}\;\cdot \;K_{\max}=8$ was found to be sufficient, where $R_{mt}$ is the smallest atomic sphere radius in the unit cell and $K_{\max}$ is the magnitude of the largest K vector. A total of 4500 *k* points were used for the self-consistent field (SCF) calculation for the tetragonal unit cell. Later, a finer *k* mesh of 50×50×15*k* points was used for the density of states (DOS) and FS calculations. A total of 1000 *k* points for the supercell were used for each calculation to provide an adequate sampling of the Brillouin zone. The supercell size was doubled to $\sqrt{2}$ × $\sqrt{2}$ × 1. We obtained the ground state through comparing the total energies of several magnetic configurations.

## 3. Results

### 3.1. Nonmagnetic Phase: Different Role of Arsenic Layers

**Figure 2:**
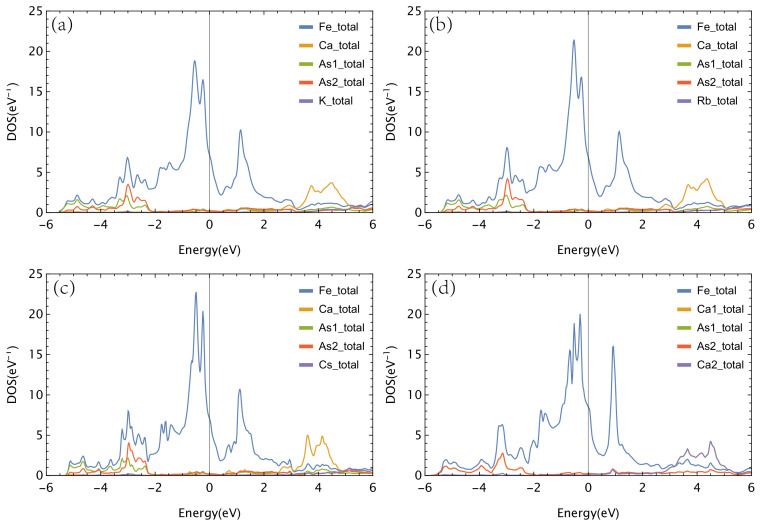
PDOS from $E_{F}-6e\;V$ to $E_{F}+6e\;V$ of nonequivalent atoms of (**a**) CaKFe${}_{4}$As${}_{4}$, (**b**) CaRbFe${}_{4}$As${}_{4}$, (**c**) CaCsFe${}_{4}$As${}_{4}$, and (**d**) CaFe${}_{2}$As${}_{2}$ (1144). Note that for CaFe${}_{2}$As${}_{2}$ (1144), Ca1 and Ca2 are equivalent, as are As1 and As2.

We show projected density of state (PDOS), results for each nonequivalent atom in Ca*A*Fe${}_{4}$As${}_{4}$ and CaFe${}_{2}$As${}_{2}$ (1144), near the Fermi energy from $E_{F}-6e\;V$ to $E_{F}+6e\;V$, in Figure 2. We set $E_{F}=0$ in the following.

We can immediately see that Fe contributes almost all the DOS near Fermi energy for all four compounds, partly due to there being four Fe atoms in a unit cell, while only one Ca, one *A*, two As1, and two As2 are partly due to dominating Fe *d* orbital contributions, which will be discussed later. For three Ca*A*Fe${}_{4}$As${}_{4}$ compounds, their DOS curves have quite similar characteristics.

- A double peak of Fe DOS in [ −1 eV, 0 eV] range.
- A single peak of Fe DOS in [ 1 eV, 2 eV].
- Ca DOS has a bump around 3 eV to 5 eV.
- The hybridization between As and Fe is clearly in [ −4 eV, −2 eV] range with coincident Fe and As peak.
- The three cases of A = K, Rb, and Cs have negligible DOS contributions in this [ −6 eV, 6 eV] whole range except for a small rising tail near 6 eV.

However, several feature tendencies move, from K to Cs, in increasing ionic radius order.

- The double peak of Fe DOS increases, while the single peak of Fe DOS decreases from K to Cs.
- The Ca DOS bump becomes narrower from K to Cs.
- The hybridization peaks of As and Fe become broader from K to Cs.

One particular thing is that As2 hybridizes significantly more with Fe than As1 in Ca*A*Fe${}_{4}$As${}_{4}$; as we can see, the As2 peak is almost twice of the As1 peak. However, As2 is further away from the Fe plane than As1 by about 0.05 Å for CaKFe${}_{4}$As${}_{4}$ and CaCsFe${}_{4}$As${}_{4}$, and 0.03 Å for CaRbFe${}_{4}$As${}_{4}$. We can prove that this enhancement of As2–Fe hybridization results from a divalent cation Ca^2^+ replaced by a monovalent cation *A*^+^.

**Figure 3:**
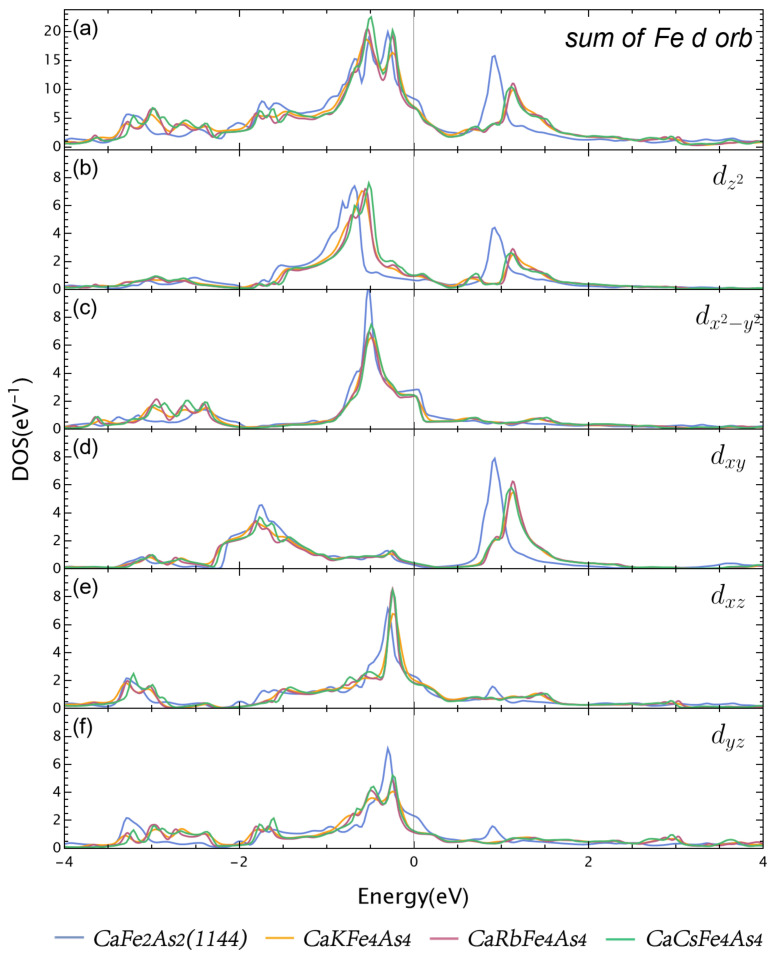
(**a**) PDOS of Fe *d* orbital of CaFe_2_As_2_ (1144) and Ca*A*Fe${}_{4}$As${}_{4}$ (*A* = K, Rb, and Cs). (**b**–**f**) PDOS of Fe $d_{z^{2}}$, $d_{x^{2}-y^{2}}$, $d_{xy}$, $d_{xz}$, $d_{yz}$ for CaFe${}_{2}$As${}_{2}$ (1144) and Ca*A*Fe${}_{4}$As${}_{4}$ (*A* = K, Rb, and Cs), and y-axis range isthe same for (**b**–**f**).

For CaFe${}_{2}$As${}_{2}$ (1144), note that Ca1 and Ca2 are equivalent as in CaFe${}_{2}$As${}_{2}$’s original symmetry, and so are As1 and As2. The features of Ca and As DOS in CaFe${}_{2}$As${}_{2}$ (1144) are similar to those in Ca*A*Fe${}_{4}$As${}_{4}$. However, the profile of Fe DOS has some obvious differences between CaFe${}_{2}$As${}_{2}$ (1144) and Ca*A*Fe${}_{4}$As${}_{4}$. The Fe DOS peak at 1 eV shifts left about 0.2 eV and is much higher and narrower than that in Ca*A*Fe${}_{4}$As${}_{4}$. The Fe DOS is also becoming slightly higher around the Fermi energy than Ca*A*Fe${}_{4}$As${}_{4}$. We show the PDOS of Fe *d* orbitals for all four compounds in Figure 3.

The local projection coordinate system is the same as the axis of the crystal unit cell. First, note that the $d_{xz}$ and $d_{yz}$ are equivalent in the original CaFe${}_{2}$As${}_{2}$ symmetry, and they become nonequivalent due to the nonequivalent of *A* and Ca atoms in 1144 symmetry. As shown in Figure 3, the partial DOS profiles of $d_{xz}$ and $d_{yz}$ in Ca*A*Fe${}_{4}$As${}_{4}$ are quite different, and especially, the DOS of $d_{yz}$ is significantly suppressed to about half of $d_{xz}$ at the Fermi energy. This indicates that although the *A* atom almost has no contribution to DOS at Fermi energy, it implicitly passes its effect through As and finally to the Fe $d_{xz}$ and $d_{yz}$. Second, the DOS peak of Fe around 1 eV is mainly contributed by $d_{z^{2}}$ and $d_{xy}$, and the peaks almost coincide. On the other hand, the peaks of Ca*A*Fe${}_{4}$As${}_{4}$ are shifted to the right relative to CaFe${}_{2}$As${}_{2}$ (1144). Two peaks between −1 eV to 0 eV in Fe DOS are from $d_{z^{2}}$, $d_{x^{2}-y^{2}}$, $d_{xz}$, and $d_{yz}$, respectively. The $d_{z^{2}}$ part shows the most discrepancy between CaFe${}_{2}$As${}_{2}$ and Ca*A*Fe${}_{4}$As${}_{4}$. Finally, the DOS at Fermi energy is contributed mainly by $d_{x^{2}-y^{2}},d_{xz},d_{yz}$.

### 3.2. Electronic Structure: CaAFe_4_As_4_ versus CaFe_2_As_2_ (1144)

**Figure 4:**
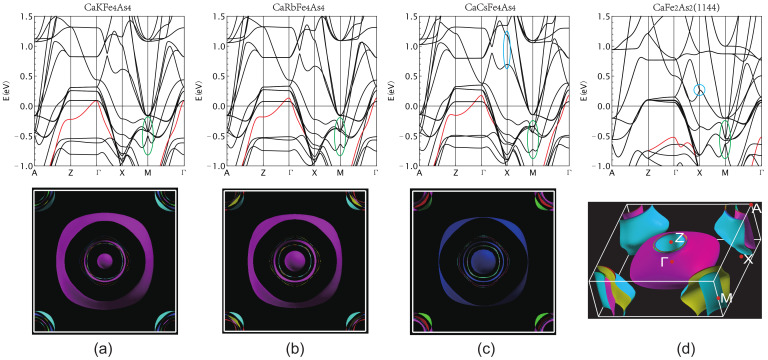
Electronic band structure along high-symmetry path A−Z−Γ−X−M−Γ and Fermi surface topology of (**a**) CaKFe${}_{4}$As${}_{4}$, (**b**) CaRbFe${}_{4}$As${}_{4}$, (**c**) CaCsFe${}_{4}$As${}_{4}$, and (**d**) CaFe${}_{2}$As${}_{2}$ (1144).

The electronic band structure and the corresponding Fermi surface topology of Ca*A*Fe${}_{4}$As${}_{4}$ (*A* = K, Rb, and Cs) and CaFe${}_{2}$As${}_{2}$ (1144) are shown in Figure 4.

Since the unit cell of CaFe${}_{2}$As${}_{2}$ (1144) is enlarged with respect to the primitive cell of CaFe${}_{2}$As${}_{2}$, the Brillouin zone (BZ) of CaFe${}_{2}$As${}_{2}$ (1144) is a folded version of the original CaFe${}_{2}$As${}_{2}$. It is this folded version of the electronic structure that makes the comparison to 1144 Ca*A*Fe${}_{4}$As${}_{4}$ meaningful. The basic feature of fermiology is that electron and hole pockets are located at *M* and Γ points, respectively. In all four compounds, there are four electron pockets at *M*. Ca*A*Fe${}_{4}$As${}_{4}$ has seven hole pockets at Γ and CaFe${}_{2}$As${}_{2}$ (1144) has six hole pockets at Γ. The one extra hole pocket that appears in 1144 Ca*A*Fe${}_{4}$As${}_{4}$ can be resolved to be the central small oblate ellipsoid Fermi pocket which is a circle in the xy plane with a short axis along the Γ–Z direction. This additional ellipsoid is one of the main differences in fermiology compared to CaFe${}_{2}$As${}_{2}$ (1144), which does not have such an ellipsoid at all. In Figure 4a–c, curves marked in red are bands that correspond to the central ellipsoid pocket. We can clearly see how the top of this hole band rises and passes the other two nearly degenerate hole bands from K to Cs at Γ point, and the corresponding ellipsoids grow bigger. The corresponding band of CaFe${}_{2}$As${}_{2}$ is located far below Fermi energy about −0.5 eV, as shown in Figure 4d, marked as a red curve. The drastic band shift results from the alkali atom replacing the Ca atom. By making band projection, as shown later, we find that this band has substantial interstitial weight, and except for dominant $d_{xz}$, $d_{yz}$ weight, there is also negligible Ca and *A* weights to this band. The second difference is degeneracy lifting. In CaFe${}_{2}$As${}_{2}$ (1144), four electron pockets around the BZ corner form two pairs, and each are degenerate on the surface of BZ, as marked by a red contour in Figure 4d. The degeneracy is lifted in Ca*A*Fe${}_{4}$As${}_{4}$ and electronic pockets are more cylindrical. For hole pockets, there is similar behavior. The outermost hole pocket is much more 3D-like than those in Ca*A*Fe${}_{4}$As${}_{4}$. The contours of hole pockets on the top face of BZ in Ca*A*Fe${}_{4}$As${}_{4}$ are much more separated than those of CaFe${}_{2}$As${}_{2}$ (1144), especially the outermost hole pocket, which can be viewed as a kind of "degeneracy lifting". The inner pockets in CaFe${}_{2}$As${}_{2}$ (1144) are not visible in Figure 4d and are quite cylindrical but not as perfect as those in Ca*A*Fe${}_{4}$As${}_{4}$. The degeneracy lifting effect can also be clearly seen in the band structure along the line A−Z marked by green circles in Figure 4a–d.

**Figure 5:**
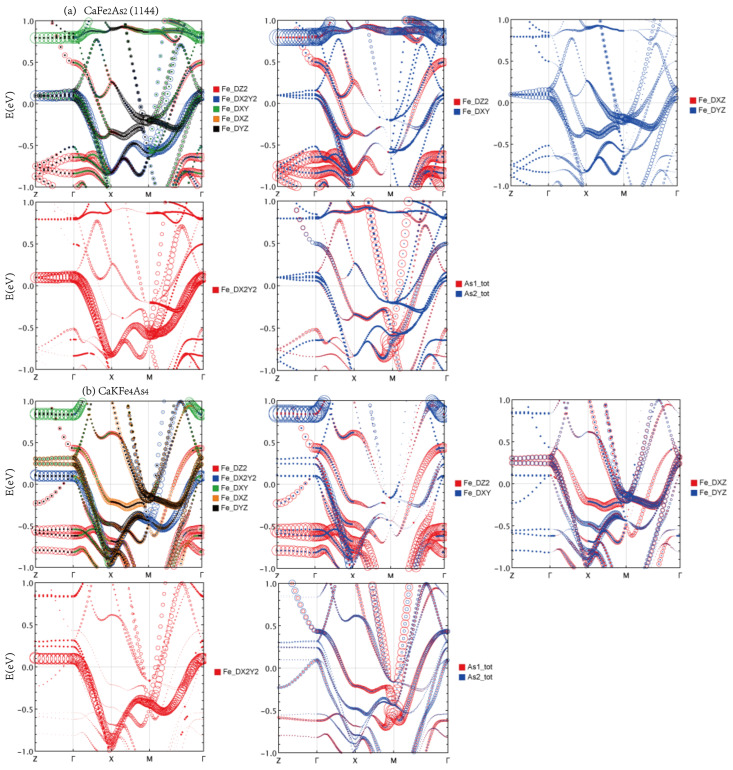
Electronic bands projected in various orbital contents for (**a**) CaFe${}_{2}$As${}_{2}$ (1144) and (**b**) CaKFe${}_{4}$As${}_{4}$. The size of the circle represents the weight of the orbital at a particular *k* point. To make a better illustration, the circle size is not on the same scale between figures. It is only meaningful to compare the relative circle size within one figure.

A plot along the Γ−M−Γ path shows that the dispersion band in CaFe${}_{2}$As${}_{2}$ (1144) is similar to Dirac at −0.6 eV, except that it (must be symmetric) disperses nearly quadratically away from the *M* point. However, this band propagates in two directions in the plane. The point at *M* is a van Hove singularity with two normal negative masses, m${}_{x}$ = m${}_{y}$, and a minimal small positive mass along the k${}_{z}$ direction, so the band quickly becomes linear (massless). This van Hove singularity, 0.6 eV lower than E${}_{F}$, does not affect the low-energy properties, including our expected superconductivity. We find that as half of the Ca atoms are replaced by K atoms, the corresponding van Hove singularity shifted to −0.4 eV in CaKFe${}_{4}$As${}_{4}$, but disappeared in CaRbFe${}_{4}$As${}_{4}$ and CaCsFe${}_{4}$As${}_{4}$. From Figure 5, it can be seen that this energy band is mainly contributed by the As1 atom. Due to the replacement of the Ca layer in CaFe${}_{2}$As${}_{2}$ (1144), the original two As atoms are different. The existence of this energy band reflects the very unusual influence of the As1 atom on this structure.

To resolve the details of various orbital contents of the band structure, we also show diverse projected band structures to support our analysis in Figure 5. Since Ca*A*Fe${}_{4}$As${}_{4}$ has a similar band structure, we chose CaKFe${}_{4}$As${}_{4}$ as the typical 1144 type to compare with CaFe${}_{2}$As${}_{2}$ (1144). Note that the circle size, which represents partial orbital weight, is not on an identical scale across figures. It is only meaningful to compare the relative size of the circle within each figure.

In Figure 5, these four electron dispersions consist of almost $d_{xz}$ and $d_{yz}$, which are equivalent in CaFe${}_{2}$As${}_{2}$ (1144) due to symmetry, as we mentioned before. The degeneration is reasonably lifted due to As1 and As2 nonequivalence caused by the *A* atom in 1144. The blue circle indicates that one of the pairs of bands significantly shifted upward and split. Finally, except for the outermost hole pocket and the innermost hole pocket in Ca*A*Fe${}_{4}$As${}_{4}$, all other hole pockets are excellent cylinders in Ca*A*Fe${}_{4}$As${}_{4}$ and CaFe${}_{2}$As${}_{2}$ (1144). The outermost hole pocket becomes more cylindrical from K to Cs. However, it is very 3D-like in CaFe${}_{2}$As${}_{2}$ (1144). Moreover, the electron pockets around *M* are also much more 3D-like than those in Ca*A*Fe${}_{4}$As${}_{4}$.

We now analyze the projected band structure, especially the *d* orbitals. The DOS of $d_{z^{2}}$ and $d_{xy}$ are below the Fermi energy, and the corresponding band projections are quite similar for Ca*A*Fe${}_{4}$As${}_{4}$ and CaFe${}_{2}$As${}_{2}$ (1144). We can see that $d_{z^{2}}$ contributes mainly to the outermost hole pockets, thus resulting in the more 3D-like feature of this pocket. $d_{xy}$ contributes mainly to inner hole pockets. The projection of $d_{x^{2}-y^{2}}$ is also similar for Ca*A*Fe${}_{4}$As${}_{4}$ and CaFe${}_{2}$As${}_{2}$ (1144). It contributes mostly to the innermost two-hole pockets in CaFe${}_{2}$As${}_{2}$ (1144), which are the nearest two-hole pockets to the central ellipsoid in Ca*A*Fe${}_{4}$As${}_{4}$. The most crucial difference lies in $d_{xz}$ and $d_{yz}$, as mentioned above. They are equivalent in CaFe${}_{2}$As${}_{2}$ (1144). However, it is drastically different in Ca*A*Fe${}_{4}$As${}_{4}$. First, the $d_{xz}$ contributes to the outermost hole pockets significantly, while $d_{yz}$ contributes almost none at all. All other hole pockets show roughly equal $d_{xz}$ and $d_{yz}$ components; however, for electron pockets, it is a little complicated. Along the *k* path M−Γ, $d_{xz}$ and $d_{yz}$ only contribute equally in two of the four bands, while the other two do not contribute. Along the M−X direction, in each pair of bands, one is mainly $d_{xz}$ and the other is mainly $d_{yz}$. We can see similar redistribution behavior of As1 and As2 weights in the electron pockets, as shown in Figure 5. Near $E_{F}$, mainly As *p* orbitals come into play, especially for the inner two electron pockets, which also receive the most As weight. The most significant hole pocket also has a substantial As *s* orbital contribution. In Ca*A*Fe${}_{4}$As${}_{4}$, the weight distributions are quite different for As1 and As2 near $E_{F}$. The largest hole pocket has roughly the same weight as As1 and As2, and all other hold pockets are more weighted in As2. For the electron bands along M−X, the weight distribution of As1 and As2 is reversed inside each pair of bands and is more drastically separated in the inner pair, especially the innermost band, which is exclusively occupied by As1. For electron bands along M−Γ, the innermost band is also much more weighted by As1, all other bands are less weighted, and As1 and As2 are roughly equal.

### 3.3. Fermi Surface Nesting

A prevailing theory for iron-based superconductors with both electron and hole pockets is that good FS nesting leads to magnetic spin fluctuation in the mother phase, and doping will suppress this pair-breaking magnetic fluctuation; thus, superconductivity emerges [26]. Fermi surface instabilities are analyzed in terms of the electronic susceptibility $\chi_{0}\left(q\right)$

(1) $$\chi_{0}\left(q\right)=\frac{1}{N}\sum_{k,m,n}{\left|M_{kn,k+qm}\right|}^{2}\frac{f\left(\varepsilon_{k+q,m}\right)-f\left(\varepsilon_{k,n}\right)}{\varepsilon_{k+q,m}-\varepsilon_{k,n}+i\eta }$$

where $M_{kn,k^{\prime }m}$ is a matrix element of $\exp\left(iq\;\cdot \;r\right)$ between Bloch functions. We will evaluate $\chi_{0}\left(q\right)$ without the matrix element, called “generalized susceptibility”. Peaks in the real part of $\chi_{0}\left(q\right)$ indicate the wave vector q instabilities. The structure in $\chi_{0}\left(q\right)$ arises from Fermi surface nesting near $E_{F}$.

**Figure 6:**
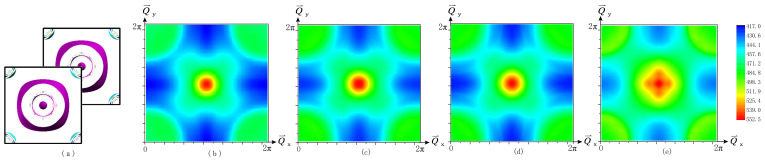
Panel (**a**) shows top view of Fermi surfaces sheets of CaKFe${}_{4}$As${}_{4}$. Each figure contains an additional copy of the Fermi surface displaced by Q=(π,π,0). The q-space plot of the general susceptibility χ(q) of (**b**) CaKFe${}_{4}$As${}_{4}$, (**c**) CaRbFe${}_{4}$As${}_{4}$, (**d**) CaCsFe${}_{4}$As${}_{4}$, and (**e**) CaFe${}_{2}$As${}_{2}$ (1144), as described in the text.

We analyze the FS topology in the nonmagnetic phase. The top view of the Fermi surface sheets of Ca*A*Fe${}_{4}$As${}_{4}$ is shown in Figure 6b–d with a copy displaced by $Q=\left(\pi ,\pi ,0\right)$ to show the geometric overlap of electron and hole pockets, and we also calculate the FS topology of CaFe${}_{2}$As${}_{2}$ (1144) as a comparison, as shown in Figure 6e. The plot also covers the entire zone, with the plot centered at $Q=\left(\pi ,\pi ,0\right)$. Γ points lie at the corners, indicating that short-range AFM fluctuations may provide the superconducting pairing mechanism.

The outermost hole pocket is the most 3D-like pocket. However, this pocket is too large to participate in the nesting process. Nesting occurs mainly between inner hole pockets at Γ and all-electron pockets at *M*. This is consistent with the real part of general susceptibility, which shows the highest peak at $Q=\left(\pi ,\pi ,0\right)$.

### 3.4. Antiferromagnetic Phases

Here, we discuss the basic features and magnetic energies of the electronic structure of the stripe antiferromagnetic (SAFM) phase in Ca*A*Fe${}_{4}$As${}_{4}$ (*A* = K, Rb, and Cs) and CaFe${}_{2}$As${}_{2}$ (1144). The types of nonmagnetic (NM), ferromagnetic (FM), and SAFM ordering types are studied. The SAFM order corresponds to each Fe atom being antiparallel to its neighbors along the a and b axes, which is the second Fe neighbor. The total energy differences of FM and SAFM magnetic configurations relative to the NM phases are listed in Table 2, where the Fe atomic sphere moments are also presented.

For the SAFM phase, the moment is much higher, and the energy is more favorable than for the FM phase in Ca*A*Fe${}_{4}$As${}_{4}$ (*A* = K, Rb, Cs) and CaFe${}_{2}$As${}_{2}$ (1144). The SAFM alignment is most favorable by a large margin; 136 meV/Fe, 121 meV/Fe, 141 meV/Fe, and 364 meV/Fe lower than FM alignment in CaKFe${}_{4}$As${}_{4}$, CaRbFe${}_{4}$As${}_{4}$, CaCsFe${}_{4}$As${}_{4}$, and CaFe${}_{2}$As${}_{2}$ (1144), respectively, resulting in an SAFM ground state. The differences of Fe moments in the above phases and 1144 materials, as shown in Table 1, effectively prove that magnetism has a substantial itinerant character. The energy gain of itinerant magnets for magnetism is $I\;\cdot \;m^{2}/4$ (*I* is Stoner *I* and *m* is the moment), which is larger in the SAFM phase than in the FM phase and largest in the SAFM state of CaCsFe${}_{4}$As${}_{4}$. Thus, the energy of itinerant magnets is larger in CaCsFe${}_{4}$As${}_{4}$ than in CaKFe${}_{4}$As${}_{4}$ and CaRbFe${}_{4}$As${}_{4}$. The comparison between the magnetic moments of the Fe atoms of Ca*A*Fe${}_{4}$As${}_{4}$ (*A* = K, Rb, and Cs) in FM shows that the results obtained by us and Mai et al. [17] are almost the same.

**Figure 7:**
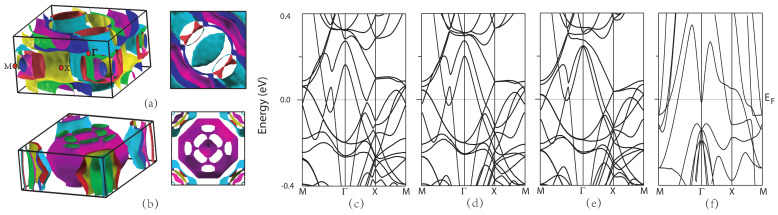
Fermi surface of (**a**) CaKFe${}_{4}$As${}_{4}$ and (**b**) CaFe${}_{2}$As${}_{2}$ (1144) and electronic band structures of (**c**) CaKFe${}_{4}$As${}_{4}$, (**d**) CaRbFe${}_{4}$As${}_{4}$, (**e**) CaCsFe${}_{4}$As${}_{4}$, and (**f**) CaFe${}_{2}$As${}_{2}$ (1144).

Figure 7 shows the FS topology and band structure of CaKFe${}_{4}$As${}_{4}$, CaRbFe${}_{4}$As${}_{4}$, and CaCsFe${}_{4}$As${}_{4}$ in the SAFM phase, and we also calculate the FS topology band structure of CaFe${}_{2}$As${}_{2}$ (1144) as a comparison. The band structure of CaFe${}_{2}$As${}_{2}$ (1144) is quite different due to the presence of alkali metals. It is shown that adding magnetics has a significant influence on the Fermi surfaces, and there is little distinct difference in FSs among CaKFe${}_{4}$As${}_{4}$, CaRbFe${}_{4}$As${}_{4}$, and CaCsFe${}_{4}$As${}_{4}$. The Fermi pocket in the center becomes larger from CaKFe${}_{4}$As${}_{4}$ to CaCsFe${}_{4}$As${}_{4}$, as shown in Figure 7b. Several d orbitals contribute to the states near the Fermi level, similar to many other iron-based superconductors. Figure 7c–e show the band dispersion of Ca*A*Fe${}_{4}$As${}_{4}$ (*A* = K, Rb, and Cs) in the SAFM order.

**Figure 8:**
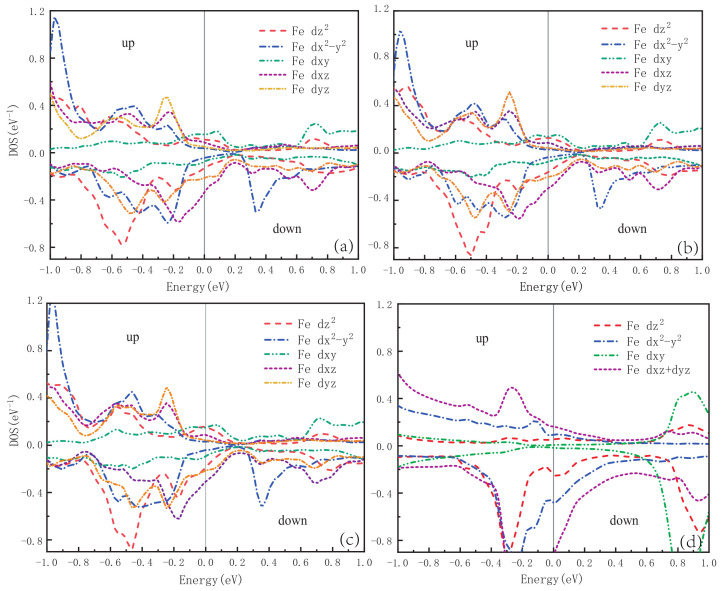
Partial densities of states (PDOS) for the Fe(up) and Fe(dn) atoms in SAFM phase of superconductors **(a**) CaKFe${}_{4}$As${}_{4}$, (**b**) CaRbF${}_{4}$As${}_{4}$, (**c**) CaCsFe${}_{4}$As${}_{4}$, and (**d**) CaFe${}_{2}$As${}_{2}$ (1144).

Figure 8a–c show the projected density of states (PDOS) for five d orbitals of Fe (spin up and down) of Ca*A*Fe${}_{4}$As${}_{4}$ (*A* = K, Rb, and Cs) in the ground state, and we also calculate the PDOS of CaFe${}_{2}$As${}_{2}$ (1144) as a comparison, as shown in Figure 8d. It is obvious that the PDOS at the Fermi level ($N(E_{F})$) is mainly contributed by Fe (spin up) $d_{xy}$ and $d_{z^{2}}$ orbitals in CaKFe${}_{4}$As${}_{4}$, CaRbFe${}_{4}$As${}_{4}$, and CaCsFe${}_{4}$As${}_{4}$. However, for Fe (spin down), PDOS shows that Fe $d_{xz}$ and $d_{yz}$ orbitals mainly contribute to the $N(E_{F})$.

## 4. Discussion and Summary

In summary, we used the full-potential linearized augmented plane wave method and GGA to study the electronic structure of 1144-type Ca*A*Fe${}_{4}$As${}_{4}$ (*A* = K, Rb, and Cs) and a unique form of 122 type CaFe${}_{2}$As${}_{2}$ with reduced 1144 symmetry, for which we have not seen a similar analysis in other articles. The 1144 symmetry of CaFe${}_{2}$As${}_{2}$ has the same BZ and orbital local projection coordinate, which makes a comparison with Ca*A*Fe${}_{4}$As${}_{4}$ meaningful. Due to the bilayer structure of FeAs in a primitive cell of 1144, we obtained as many as eleven bands and ten bands passing Fermi energy in Ca*A*Fe${}_{4}$As${}_{4}$ and CaFe${}_{2}$As${}_{2}$ (1144), respectively. We identify that the additional oblate hole ellipsoid at Γ in Ca*A*Fe${}_{4}$As${}_{4}$ originates from a band located −0.5 eV below $E_{F}$ in CaFe${}_{2}$As${}_{2}$ (1144). Moreover, this unique 3D pocket has substantial interstitial and Ca, A weights other than dominant $d_{xz}$, $d_{yz}$ weights. The nonequivalence of As1 and As2 within one FeAs layer does not appear in other iron-based superconductors 11, 1111, 111, and 122 types. They have crucial effects on electron structure: First, our study shows that the $d_{xz}$ and $d_{yz}$ orbitals, which are degenerate in CaFe${}_{2}$As${}_{2}$, become nondegenerate in Ca*A*Fe${}_{4}$As${}_{4}$ due to two nonequivalent arsenic atoms (As1 and As2). The partial DOS of $d_{yz}$ is suppressed to almost half of $d_{xz}$. The weights of $d_{xz}$ and $d_{yz}$ are drastically unequal in the outermost hole pocket and electron bands along M−X. It even shows an interesting alternating weight pattern within each pair of electron bands, with one band mainly $d_{xz}$ and the other primarily $d_{yz}$. Second, the replacement of *A* atoms will not lead to apparent changes in the FeAs conductive layer. Third, the unusual band appears in CaFe${}_{2}$As${}_{2}$ (1144) and gradually disappears in the evolution of K to Cs, which is introduced by the As1 atom and shows a strong dispersion perpendicular to the FeAs layer, suggesting that it is related to the peculiar van Hove singularity below the Fermi level.

We hope that these findings will help investigate which aspects of these differences are relevant to understanding their superconducting properties, thereby providing theoretical support for the search for superconducting materials with higher T${}_{c}$.

## Figures and Tables

**Table 1:** Structure parameters for Ca*A*Fe${}_{4}$As${}_{4}$ [23] and CaFe_2_As_2_ (1144) used in this article.

System	a(Å)	c(Å)	$z_{\begin{array}{c} \mathrm{Fe} \end{array}}$	$|z_{\begin{array}{c} \mathrm{Fe} \end{array}}-z_{{\mathrm{As}}_{1}}|$	$|z_{\begin{array}{c} \mathrm{Fe} \end{array}}-z_{{\mathrm{As}}_{2}}|$
CaFe_2_As_2_ (1144)	3.907	11.601	0.75000	0.11509	0.11509
CaKFe${}_{4}$As${}_{4}$	3.866	12.817	0.77787	0.09742	0.10150
CaRbFe${}_{4}$As${}_{4}$	3.876	13.104	0.77803	0.09452	0.09696
CaCsFe${}_{4}$As${}_{4}$	3.891	13.414	0.77899	0.09144	0.09536

**Table 2:** Total energy difference, for Ca*A*Fe${}_{4}$As${}_{4}$ (*A* = K, Rb, Cs) and CaFe${}_{2}$As${}_{2}$ (1144), of two magnetic phases including nonmagnetic (NM), ferromagnetic (FM), and striped AFM (SAFM). The reference is the energy of the NM phase (ΔE = E ${}_{(SA)FM}$ − *E*${}_{NM}$). The corresponding magnetic moment in the Fe sphere is given.

	CaKFe${}_{4}$As${}_{4}$			CaRbFe${}_{4}$As${}_{4}$			CaCsFe${}_{4}$As${}_{4}$			CaFe${}_{2}$As${}_{2}$ (1144)		
Magnetic structure	NM	FM	SAFM	NM	FM	SAFM	NM	FM	SAFM	NM	FM	SAFM
(a) Relative energy (meV)	0	−10	−146	0	−4	−125	0	1	−140	0	259	−105
(b) Fe moment ($\mu_{B}$)	0	0.44	1.18	0	0.33	1.15	0	0.55	1.20	0	1.71	1.66

## Data Availability

The data presented in this study are available on request from the corresponding author.
